# Physical health disparities and severe mental illness: A longitudinal comparative cohort study using hospital data in Northern Ireland

**DOI:** 10.1192/j.eurpsy.2023.2441

**Published:** 2023-08-14

**Authors:** Rachel McCarter, Michael Rosato, Annette Thampi, Ruth Barr, Gerard Leavey

**Affiliations:** 1 Bamford Centre for Mental Health and Wellbeing, Ulster University, Coleraine, UK; 2 Administrative Data Research – Northern Ireland (ADR-NI), Ulster University, Coleraine, UK; 3 Belfast NHS Trust, Belfast, UK

**Keywords:** bipolar disorder, morbidity, mortality, multimorbidity, severe mental illness

## Abstract

**Background:**

People with severe mental illness (SMI) die prematurely, mostly due to preventable causes.

**Objective:**

To examine multimorbidity and mortality in people living with SMI using linked administrative datasets.

**Method:**

Analysis of linked electronically captured routine hospital administrative data from Northern Ireland (2010–2021). We derived sex-specific age-standardised rates for seven chronic life-limiting physical conditions (*chronic kidney disease, malignant neoplasms, diabetes mellitus, chronic obstructive pulmonary disease, chronic heart failure, myocardial infarction, and stroke*) and used logistic regression to examine the relationship between SMI, socio-demographic indicators, and comorbid conditions; survival models quantified the relationship between all-cause mortality and SMI.

**Results:**

Analysis was based on 929,412 hospital patients aged 20 years and above, of whom 10,965 (1.3%) recorded a diagnosis of SMI. Higher likelihoods of an SMI diagnosis were associated with living in socially deprived circumstances, urbanicity. SMI patients were more likely to have more comorbid physical conditions than non-SMI patients, and younger at referral to hospital for each condition, than non-SMI patients. Finally, in fully adjusted models, SMI patients had a twofold excess all-cause mortality.

**Conclusion:**

Multiple morbidities associated with SMI can drive excess mortality. While SMI patients are younger at referral to treatment for these life-limiting conditions, their relatively premature death suggests that these conditions are also quite advanced. There is a need for a more aggressive approach to improving the physical health of this population.

## Introduction

People with severe mental illness (SMI), schizophrenia or bipolar affective disorder, die at younger ages than the general population [1, 2]. Most deaths of people with SMI are not attributable to unnatural causes, such as suicide or homicide. Therefore, they are mostly due to physical disorders, such as cardiovascular, respiratory, and infectious diseases, diabetes mellitus and cancers [3]. Comorbidities among people with SMI are well-documented in the literature [4–10]. For example, they have a twofold to threefold risk of diabetes and excess risk of pneumonia and emphysema [4]. Moreover, SMI is associated with a twofold likelihood of endocrine, cardiovascular, and respiratory diseases [10]. However, the evidence for cancers is less consistent, with some studies finding reduced rates of cancer among SMI patients, compared to other patients, but with higher risk of mortality, nevertheless [11].

Some of the explanations for poorer health and mortality may lie in problems and fragmentation in healthcare systems. Thus, a New Zealand longitudinal study found that people with mental disorders had more (and longer) hospitalisations, developed physical problems at earlier ages, and died at younger ages than those without a mental disorder [12]. The COVID-19 pandemic appears to have increased the mortality rates of SMI patients, due to pre-existing physical health problems and thus, greater susceptibility to COVID-19 infection [13]. While the side effects of psychotropic medications may contribute to the increased health vulnerability of people with SMI, other lifestyle factors such low physical exercise, poor diet, high alcohol, and cigarette use, are also contributory [14].

### Multimorbidity

Multimorbidity is generally recognised as the coexistence of multiple health conditions [15–20]. Several disease typologies for examining multimorbidity in hospital patients have been developed [16–20]. Tonelli et al. [20] used the definition of three or more morbidities. However, definitions of multimorbidity have been contested as to the number of conditions and level of chronicity [21]. Parity of esteem between mental health and physical health, and improving health outcomes for people with SMI are central to mental health policies in the UK and elsewhere [21, 22]. Thus, there is a need to explore outcomes related to major life-limiting conditions for people with schizophrenia and bipolar disorder (SMI) compared to people without SMI. This may provide better evidence on disparities, opportunities for early diagnosis, and gaps in screening and treatment.

### Aim

To examine the relationship between SMI, various life-limiting physical disorders (and co-morbidities), and mortality in a population attending at hospital settings in Northern Ireland (NI) over an 11-year period between 2010 and 2020.

### Study population

All individuals normally resident in Northern Ireland, aged 20 or more years at January 01, 2010, and registered as having a hospital admission between 2010 and 2021.

## Method

Secondary data analysis of routinely collected electronically linked hospital administrative records: an initial study population comprising persons normally resident in Northern Ireland and registered with a General Practitioner at January 01, 2010 (the study spine); linked with hospitals admissions data (2010–2021); an indicator of small area-level social deprivation at 2010 (NIMDM); and all-cause mortality information. These constituent databases are managed and maintained by the *Honest Broker Service*, part of the Northern Ireland Health and Social Care Business Services Organisation [23].

To choose the chronic conditions, we adopted a pragmatic approach: using the 30 morbidities developed by Tonelli and colleagues, we identified the seven most prevalent conditions in the study population that *inhibited the activities of daily living.* Whereas Tonelli included non-metastatic and blood cancers, we included all cancers. Each hospital admission record included both a clinical definition (ICD-10 coding) of the primary diagnosis and up to 15 associated secondary diagnoses – from these the conditions were codified as binary fields (0 = not present/1 = present) in each study year separately: *myocardial infarction* (I21.9), *stroke* (I63.9), *coronary heart failure* (I50.9), *chronic kidney disease* (N18.9), *chronic obstructive pulmonary disease – COPD* (J44.9), *diabetes* (E08–E13), and *malignant neoplasms* (C01–087). Because of the emphasis on the number of coexistent conditions, in this study, no distinction is made between primary and secondary diagnosis. Again, for each year, the presence of each condition was accumulated forming an indicator of multimorbidity – the number of conditions each hospital patient recorded (none, one, two, three, and four or more). Also, worthy of note – some individuals included in the hospital population at each year will have reported conditions not included in the sentinel list above. Consistent with other research [2, 24], we also derived *schizophrenia* (F20.0–20.9 excluding F20.7) and *bipolar disorder* (F31), with both combined for analysis (SMI – *serious mental illness*). These were defined similarly to the physical health conditions noted above and formed the main outcome indicators for analysis.

Socio-demographic characteristics included: two forms of age group (in 10-year age bands – age group in 2010, and age-group at each year between 2010 and 2021; gender (male or female); locale of residence (rural, intermediate, or urban); and finally, an indicator of area-level deprivation – the Northern Ireland Multiple Deprivation Measure (NIMDM) – derived as deciles and ordered from least most deprived.

### Analysis

Statistical analyses were performed using Stata/SE version 17.0 [25]. Frequencies and associated percentages are provided for all variables. To reflect the ageing of the population sex-specific age-standardised rates (ASR; per 1,000 population) were derived separately by year of follow-up for each physical health condition (and number of conditions) As is usual practice in deriving ASR, 5-year age groups were used. For brevity, and clarity, and to reflect the trajectory of this population ageing, rates derived for the first year, mid-point (2015) and end-point (2020) of the study are presented (intermediate years are available on request). The characteristics associated with occurrence of SMI, individual morbidities and multiple conditions are quantified using logistic regression (again, stratified by year). Wilcoxon rank-sum tests were used to compare the median differences between SMI and non-SMI patients for age at first referral to hospital services. We also analysed these differences for sex, rurality, and area deprivation. Finally, the relationship between SMI, all-cause mortality, the physical health conditions and the general socio-demographic circumstance is assessed using Cox Proportional Hazard models in a set of four incrementally adjusted models, with age modelled as a continuous variable.

## Results

The hospital population between 2010 and 2021 comprised 929,412 patients – 413,397 (44.5%) male and 516,015 (55.5%) female (Table 1). Of these, 10,965 (1.18%) patients recorded a SMI – 5,317 (48.5%) male and 5,648 (51.5%) female. For those with an SMI diagnosis, the mean ages were 48.3 (SD = 16.4) and 53.8 (SD = 17.5) years, respectively, for males and females – this compares with 50.8 (SD = 17.2) and 49.3 (SD = 19.0) for non-SMI patients. While the non-SMI population was distributed evenly across the deprivation decile bands (approximately 10% each), a social gradient is noticeable among the SMI group, with increasing levels of deprivation – from 6.8% in the least to 16.4% in the most deprived decile. SMI patients were more likely than non-SMI patients to live in urban settings (28.7% vs. 20.5%).

**Table 1.** Socio-demographic characteristics associated with persons recording a diagnosis of SMI

	Non-SMI	SMI
Age at 2010 (mean and standard deviation), by gender and broad age bands		
Male		
20–44	33.0 (7.3)	32.9 (7.4)
45–64	49.0 (2.6)	48.9 (2.6)
65 plus	73.7 (6.6)	72.6 (6.0)
All 20 plus	50.8 (17.2)	48.3 (16.4)
Female		
20–44	31.6 (7.2)	33.5 (7.3)
45–64	49.0 (2.6)	48.8 (2.6)
65 plus	75.3 (7.2)	74.8 (6.7)
*All 20 plus*	49.3 (19.0)	53.8 (17.5)
	***n* (%)**	***n* (%)**
Male	408,080 (44.4)	5,317 (48.5)
Female	510,367 (55.6)	5,648 (51.5)
*All*	918,447 (100)	10,965 (100)
Rural	242,556 (26.7)	1,930 (17.7)
Intermediate	478,765 (52.8)	5,888 (53.7)
Urban	186,161 (20.5)	3,147 (28.7)
Northern Ireland multiple deprivation measure (NIMDM): area-level social deprivation (as at 2010)		
1: least deprived	83,501 (9.2)	774 (6.8)
2	82,793 (9.1)	662 (6.0)
3	90,373 (10.0)	847 (7.7)
4	92,680 (10.2)	964 (8.8)
5	89,091 (9.82)	935 (8.5)
6	97,287 (10.7)	1131 (10.3)
7	98,016 (10.8	1194 (10.9)
8	91,550 (10.1)	1273 (11.6)
9	92,540 (10.2)	1421 (13.0)
10: most deprived	89,651 (9.9)	1479 (16.4)

### Physical health morbidity

Table 2 shows sex-specific ASR (per 1,000 of the population) for the selected chronic conditions for males and females aged 20 years or more in the selected year. Overall, the most prevalent of these were *chronic kidney disease* (16.2 and 14.4% for males and females, respectively); *malignant neoplasms* (15.6 and 11.7%); *diabetes mellitus* (12.6 and 8.2%); and *COPD* (9.1 and 7.3%), with the remaining circulatory diseases featuring less prominently. A similar pattern was observed in the cause-specific ASR, which increases as the population ages over the follow-up period. ASR associated with *no stated conditions* (as expected) reduce over time, whereas those associated with the accumulating conditions increase (e.g., females recording one condition shows ASR = 178 (95% CI = 175, 180); ASR = 186 (183, 188); and ASR = 222 (219, 225) per 1,000 of the population for 2010, 2015, and 2020, respectively). Of the outcome measures – overall SMI prevalence was 1.4% (males) and 1.2% (females), with male ARS rising from 8.5 (7.9, 9.2) in 2010 to 14 (13.1, 14.9) in 2020, and female ARS rising from 5.9 (5.5, 6.3) in 2010 to 11.3 (10.7, 12.0).

**Table 2.** *Sex-specific age-standardised rates* for all chronic conditions recorded at hospital admission, by year (2010, 2015, and 2020)

Male				
	2010	2015	2020	All years
Hospital population Proportion of the 2010 population	*n* = 91,176 (100.0%) ***ASR (95% CI)***	*n* = 97,880 (107.4%) ***ASR (95*** ***% CI)***	*n* = 74,412 (81.6%) ***ASR (95% CI)***	*n* = 408,080 ***n* (%)**
Chronic kidney disease	58 (56.6, 59.6)	93 (91.8, 95.1)	113 (110.8, 114.9)	66,302 (16.2)
Malignant neoplasms	74 (72.3, 75.5)	80 (78.6, 81.7)	100 (98.5, 102.3)	63,746 (15.6)
Diabetes mellitus	82 (80.1, 83.5)	96 (94.5, 97.9)	123 (121.1, 125.4)	51,504 (12.6)
COPD	48 (47.2, 49.8)	59 (57.4, 59.9)	64 (62.4, 65.4)	37,281 (9.1)
Chronic heart failure	35 (33.7, 36.0)	38 (36.6, 38.7)	48 (46.8, 49.4)	29,475 (7.2)
Myocardial infarction	20 (19.1, 20.8)	18 (17.4, 19.0)	21 (20.0, 22.0)	22,181 (5.4)
Stroke	18 (17.0, 18.6)	17 (16.1, 17.6)	21 (20.1, 22.0)	20,060 (4.9)
Number of conditions				
0	758 (755, 760)	727 (724, 729)	668 (665, 671)	na
1	178 (175, 180)	186 (183, 188)	222 (219, 225)	
2	47 (46, 48)	61 (60, 62)	76 (75, 78)	
3	14 (13, 15)	20 (19.5, 21.1)	26 (25, 27)	
4–7	4 (3.6, 4.4)	6.3 (5.8, 6.7)	7.4 (6.9, 7.9)	
Schizophrenia	7.0 (6.5, 7.6)	8.5 (7.9, 9.1)	12 (11.2, 13.1)	4,133 (1.0)
Bipolar disease	1.8 (1.5, 2.1)	2.6 (2.2, 2.9)	3.7 (3.2, 4.2)	1,427 (0.4)
All serious mental illness (SMI)	8.5 (7.9, 9.2)	10.2 (9.6, 10.9)	14.0 (13.1, 14.9)	5,319 (1.4)^£^
Female				
Hospital population Proportion of the 2010 population	*n* = 129,614 (100.0%)	*n* = 132,051 (101.9%)	*n* = 95,958 (74.0%)	*n* = 510,367
Chronic kidney disease	40 (39, 41)	84 (82.4, 85.1)	108 (106.0, 109.5)	73,483 (14.4)
Malignant neoplasms	57 (56, 59)	61 (59.4, 61.9)	80 (78.4, 81.7)	59,554 (11.7)
Diabetes mellitus	57 (56, 58)	66 (65, 67)	86 (83.9, 87.2)	41,831 (8.2)
COPD	41 (40, 42)	55 (54, 56)	62 (60.3, 63.1)	37,083 (7.3)
Chronic heart failure	27 (26, 28)	31 (30.5, 32.2)	37 (35.6, 37.6)	29,555 (5.8)
Myocardial infarction	10.2 (9.6, 10.7)	8.9 (8.5, 9.4)	8.8 (8.3, 9.4)	12,761 (2.5)
Stroke	16 (15.5, 16.8)	14 (13.5, 14.7)	17 (16.4, 17.9)	21,038 (4.1)
Number of conditions				
0	812 (810, 814)	773 (771, 775)	722 (719, 725)	na
1	144 (142, 145)	160 (158, 162)	191 (188, 193)	
2	34 (33, 35)	48 (47, 50)	63 (62, 65)	
3	9 (8.0, 9.1)	15 (14, 16)	20 (19, 20)	
4–7	2.2 (1.9, 2.4)	3.5 (3.2, 3.8)	4.4 (4.0, 4.8)	
Schizophrenia	3.6 (3.2, 3.9)	4.4 (4.0, 4.8)	6.1 (5.6, 6.6)	3,333 (0.7)
Bipolar disease	2.5 (2.2, 2.8)	3.8 (3.5, 4.1)	5.5 (5.0, 6.0)	2,638 (0.5)
All serious mental illness (SMI)	5.9 (5.5, 6.3)	8.2 (7.7, 8.7)	11.3 (10.7, 12.0)	5,648 (1.2)^£^

*Note:* Data represents (a) rates per 1,000 of the annual hospital population and 95% confidence intervals; and (b) numbers and proportions of each condition recorded.

£ Discrepancy in SMI totals is because 566 patients recording both bipolar disorder and schizophrenia.

Age standardised rates are in bold.

### SMI and the selected chronic health conditions

Table 3 shows odds ratios (ORs) associated with SMI and each of the (separately included) chronic conditions, again by indicative year of study follow-up: *kidney disease*, *COPD,* and *diabetes mellitus* maintain a more than twofold likelihood of association with SMI across the selected years; with *chronic heart failure* showing a declining likelihood (OR = 1.87: 95% CI = 1.48, 2.36; OR = 1.59: 1.30, 1.94; and OR = 1.30: 1.08, 1.57, respectively). Although the risks for stroke and myocardial infarction were raised, these were non-significant. Across all years, there was a consistent likelihood associated with cancers for SMI (in 2020, OR = 0.73 (0.62, 0.86)).

**Table 3.** Association between *serious mental illness* and the seven selected co-morbidities

	Logistic regression – relationship between SMI and selected co-morbidities, with each condition modelled separately			Median age of patient when condition first recorded in the hospital record			
	2010	2015	2020	Male		Female	
	OR (95% CI)^$^	OR (95% CI)^$^	OR (95% CI)^$^	Non-SMI	SMI	Non-SMI	SMI
Kidney disease	2.18	2.47	2.07	73 (73, 73)	66 (65, 67)	76 (76, 76)	71 (70, 72)
	(1.83, 2.61)^***^	(2.19, 2.79)^***^	(1.85, 2.32)^***^	*z* = 1.4113 (*p* = 0.000)		*z* = 12.121 (*p* = 0.0000)	
Diabetes mellitus	2.06	2.01	1.98	68 (68, 68)	60 (59, 61)	70 (70, 70)	64 (64, 66)
	(1.73, 2.49)^***^	(1.78, 2.28)^***^	(1.77, 2.11)^***^	*z* = 16.010 (*p* = 0.000)		*z* = 11.39 (*p* = 0.000)	
COPD	2.57	2.75	2.20	71 (71, 71)	62 (61, 63)	70 (70, 70)	63 (61, 64)
	(2.17, 3.04)^***^	(2.42, 3.12)^***^	(1.94, 2.50)^***^	*z* = 20.111 (*p* = 0.000)		*z* = 15.151 (*p* = 0.000)	
Chronic heart failure	1.87	1.59	1.30	76 (76, 76)	69 (67, 71)	81 (81, 81)	75 (73, 76)
	(1.48, 2.36)^***^	(1.30, 1.94)^***^	(1.08, 1.57)*	*z* = 10.614 (*p* = 0.000)		*z* = 11.737 (*p* = 0.000)	
Myocardial infarction	1.32	1.25	0.78	67 (67, 68)	65 (62, 68)	76 (76, 77)	72 (70, 76)
	(0.92, 1.89)	(0.91, 1.73)	(0.54, 1.13)	*z* = 3.755 (*p* = 0.0002)		*z* = 3.630 (*p* = 0.0003)	
Stroke	1.18	1.26	1.09	72 (72, 72)	66 (63, 68)	78 (77, 79)	74 (72, 76)
	(0.82, 1.69)	(0.93, 1.72)	(0.82, 1.45)	*z* = 7.565 (*p* = 0.000)		*z* = 5.115 (*p* = 0.000)	
Malignant neoplasms	0.74	0.82	0.73	71 (70, 71)	66 (65, 67)	68 (68, 68)	66 (65, 68)
	(0.58, 0.93)*	(0.69, 0.98)*	(0.62, 0.86)^***^	*z* = 9.325 (*p* = 0.000)		*z* = 2.835 (*p* = 0.004)	

*Note*: Data represents (a) fully adjusted logistic regression models for selected years over the study period; and (b) median age of patients when condition first recorded.

$ Logistic regression models are fully adjusted for age, gender, small-area deprivation and locale of residence.

*** *p* < 0.001.

** *p* < 0.005.

* *p* < 0.05.

We examined the relationship between an SMI diagnosis and selected socio-demographic characteristics (Table 4). SMI is more strongly associated with being male, and at younger age bands, peaking at 40–49 years and then declining. It is also associated with areas of higher social deprivation (e.g., OR = 1.07: 1.05, 1.09 in 2015) and, compared to persons living in rural areas, with living in urban or intermediate areas (OR = 2.06: 1.80, 2.36; and OR = 1.54: 1.36, 1.74, respectively). Finally, ORs associated with multimorbidity, all show increased likelihoods for people with SMI, most notably for example in 2015 – OR = 2.14 (1.92, 2.39); 3.14 (2.70, 3.66); 2.41 (1.83, 3.18); and 3.52 (2.30, 5.39) for one, two, three, and four conditions, respectively. The relationship between these central results and the study start- and end-points may reflect both the ageing of the population (increasing by 10 years over the study period) and the mortality associated with an ageing population.

**Table 4.** Socio-demographic and clinical characteristics associated with the diagnosis of *serious mental illness* (SMI) in a NI hospital population

	2010 OR (95% CI)	2015 OR (95% CI)	2020 OR (95% CI)
Number of diagnosed conditions			
0	1.00	1.00	1.00
1	1.79 (1.58, 2.04)^***^	2.14 (1.92, 2.39)^***^	1.74 (1.57, 1.94)^***^
2	2.20 (1.78, 2.73)^***^	3.14 (2.70, 3.66)^***^	2.45 (2.13, 2.82)^***^
3	3.29 (2.37, 4.56)^***^	2.41 (1.83, 3.18)^***^	2.01 (1.58, 2.56)^***^
4	1.70 (0.75, 3.82)	3.52 (2.30, 5.39)^***^	1.71 (1.05, 2.78)*
Age group at year			
20–29	0.53 (0.43, 0.65)^***^	0.62 (0.52, 0.75)^***^	0.74 (0.61, 0.90)^**^
30–39	0.68 (0.56, 0.81)^***^	0.75 (0.64, 0.89)^**^	0.77 (0.64, 0.92)^**^
40–49	1.00	1.00	1.00
50–59	0.81 (0.69, 0.96)*	0.81 (0.70, 0.93)^**^	0.96 (0.83, 1.12)
60–69	0.72 (0.61, 0.85)^***^	0.65 (0.56, 0.75)^***^	0.69 (0.59, 0.80)^***^
70–79	0.50 (0.42, 0.61)^***^	0.52 (0.45, 0.61)^***^	0.59 (0.50, 0.69)^***^
80+	0.44 (0.35, 0.54)^***^	0.36 (0.30, 0.43)^***^	0.34 (0.28, 0.41)^***^
Gender			
Male	1.00	1.00	1.00
Female	0.73 (0.66, 0.81)^***^	0.87 (0.80, 0.95)^**^	0.87 (0.80, 0.95)^**^
Area-level deprivation (least to most deprived)^a^	1.07 (1.05, 1.09)^***^	1.07 (1.05, 1.09)^***^	1.07 (1.06, 1.09)^***^
Locale of residence			
Rural	1.00	1.00	1.00
Intermediate	1.63 (1.41, 1.89)^***^	1.54 (1.36, 1.74)^***^	1.47 (1.30, 1.65)^***^
Urban	2.05 (1.74, 2.41)^***^	2.06 (1.80, 2.36)^***^	1.86 (1.63, 2.12)^***^

a Data represents odds ratios and 95% confidence intervals from fully adjusted logistic regression models for selected years covering the follow-up period.

*** *p* < 0.001.

** *p* < 0.005.

* *p* < 0.05.

### Age at first hospital referral

At first contact with hospital services for all seven physical health conditions, SMI patients were significantly younger than non-SMI patients with the same condition (Table 3). For example, COPD: for males ages 71 and 62 years for non-SMI and SMI patients, respectively (*z* = 20.111, *p* = 0.000), and similarly for females – ages 70 and 63 years (*z* = 15.151, *p* = 0.000); chronic heart failure – male = ages 76 and 69 years (*z* = 10.614, *p* = 0.000), and females = ages 81 and 75 years (*z* = 11.737, *p* = 0.000); and kidney disease – males = ages 73 and 66 years for SMI and non-SMI patients, respectively (*z* = 14.111, *p* = 0.000), and females = ages 76 and 71 years (*z* = 12.121, *p* = 0.000), respectively. The median age differences at first hospital contact for the seven conditions between SMI and non-SMI patients was between 2 (males, myocardial infarction) and 9 years (males, COPD). Stratified by sex, significant median differences remained for males and females in all conditions but were much more pronounced for male SMI patients.

### SMI, physical health multimorbidity, and all-cause mortality

Of 929,412 patients included in the study, 156,029 (16.78%) died during the follow-up period. Of those recording any SMI (10,965), 3,393 died (30.9% vs. 17.26% non-SMI). Table 5 shows the results of a series of incrementally adjusted models examining the relationship between SMI, multimorbidity, small area-level deprivation, locale of residence and all-cause mortality: with the exception of M2 – adjusted only for number of chronic physical conditions recorded (HR = 2.43: 95% CI = 2.42, 2.44) – those recording a diagnosis of SMI show a stable twofold excess likelihood of mortality across models 1, 3, and 4 (HR = 2.09: 2.03, 2.17; HR = 2.02: 1.95, 2.09; and HR = 1.97: 1.91, 2.04, respectively). In these models, the excess likelihoods associated with the number of recorded chronic physical conditions also stabilise in M3 and M4 (HR = 1.60: 1.57, 1.59 and HR = 1.57: 1.56, 1.58, respectively). A similar pattern is also apparent in the results for both bipolar disorder and schizophrenia when examined separately.

**Table 5.** *All-cause mortality*: association with serious mental illness (SMI), physical conditions, and socio-demographic circumstance (2010–2021)

	M1: SMI only HR (95% CI)	M2: M1 + number of conditions HR (95% CI)	M3: M2 + age/sex HR (95% CI)	M4: M3 + socio-economic circumstance HR (95% CI)
SMI (ref = no)				
Yes	2.09 (2.03, 2.17)^***^	1.52 (1.47,1.57)^***^	2.02 (1.95, 2.09)^***^	1.97 (1.91, 2.04)^***^
Diagnosed conditions				
Number = 1–7^$^		2.43 (2.42, 2.44)^***^	1.60 (1.57, 1.59)^***^	1.57 (1.56, 1.58)^***^
Age				
Single years from 20+^$^			1.08 (1.08, 1.08)^***^	1.08 (1.08, 1.08)^***^
Gender				
Female (reference = male)			0.89 (0.88, 0.89)^***^	0.87 (0.87, 0.88)^***^
Area-level deprivation (most to least deprived)^$^				1.02 (1.02, 1.03)^***^
Locale of residence				
Urban				1.00
Intermediate				0.91 (0.90, 0.93)^***^
Rural				0.81 (0.80, 0.82)^***^
Schizophrenia^&^ (ref = no) Yes	2.41 (2.32, 2.51)^***^	1.73 (1.66, 1.80)^***^	2.13 (2.05, 2.22)^***^	2.07 (1.99, 2.15)^***^
Bipolar disorder^&^ (ref = no) Yes	1.51 (1.40, 1.61)^***^	1.16 (1.08, 1.24)^***^	1.70 (1.59, 1.82)^***^	1.68 (1.57, 1.80)^***^

*Note:* Data represents hazard ratios (HR) and 95% confidence intervals.

$ Variables regressed as continuous.

& Component conditions of SMI – for brevity, HRs for indicative years only are presented. Full table provided on request.

*** *P* < 0.001.

## Discussion

In this study, based on the hospital records of almost 1 million people, patients with a diagnosis of SMI were at much higher risk of multiple chronic and life-limiting conditions compared to patients without SMI. Previous prevalence estimates of physical multimorbidity in SMI patients have been recorded at between 29 and 64% [7, 8, 26–28]. In this population, after adjusting for socioeconomic and other factors, we found a threefold likelihood of having more than three or more co-morbidities (excluding the SMI diagnosis) compared to other patients. We noted a twofold likelihood of association with SMI for kidney disease, COPD, and diabetes mellitus and this was consistent across the years 2011–2020. The risk of chronic heart failure declined over the same period, possibly explained by mortality in this group. Moreover, while the odds ratios associated with stroke and myocardial infarction were raised for people with SMI, these were not statistically significant, possibly due to the rapid onset and high mortality in these specific conditions. Additionally, patient and service-related barriers may be relevant here. Thus, evidence suggests that patients with schizophrenia may be less likely to be offered examination and/or accept treatment compared to non-psychiatric patient [29]. Other evidence indicates that SMI is associated with a strikingly higher mortality after myocardial infarction, even after accounting for contributing factors. While SMI patients have the highest CVD mortality, they are less likely to obtain preventative care, with a significant disparity in the monitoring of cholesterol levels and statins prescriptions, especially in people with SMI aged 60 years and over [30].

Evidence from the Swedish nationwide myocardial infarction register noted that SMI patients were younger at first diagnosis of myocardial infarction, compared to non-SMI patients [31], and this is also reflected in our findings that people with SMI, developed all the life-limiting conditions at a significantly younger median age than other patients. Given the higher mortality in this population, we might have expected a later age presentation to specialist treatment due to pathway barriers such as low GP contact and under-recognition. However, it may be that earlier presentation denotes a more rapid onset due to increased risk factors, more aggressive symptoms, and/or earlier detection than general hospital patients due to the presence of comorbidities. The median age differences between SMI and non-SMI patients are more pronounced in men, again suggestive of higher risk exposures in males, generally. Again, previous evidence showed that patients with schizophrenia have a threefold risk of sudden cardiac death as individuals from the general population [32].

Evidence on the relationship between SMI and all cancer types together have shown conflicting results [33, 34]. Thus, previous reviews have indicated that the incidence of cancer is variable with some studies reporting a higher prevalence, while others reveal similar or lower prevalence of cancer compared to the general population. However, it may be that cancers in SMI patients are missed due to complex multimorbidity and diagnostic overshadowing and/or may be less likely to obtain screening due to the negative psychiatric symptoms. For these reasons, opportunities for timely and appropriate cancer treatment are limited in this population, resulting in significantly lower survival rates after cancer diagnosis than people without SMI. Thus, SMI patients may not have a greater incidence of cancers compared to non-psychiatric patients but have higher cancer mortality [35] due to suboptimal treatment and/or greater levels of comorbidity. It may also be the case that because patients with SMI have a shorter life expectancy, a higher proportion die from cardiovascular and other diseases before reaching the expected age of death from cancer. However, there is good evidence from elsewhere that this may not be a sufficient explanation [36]. In the current study, SMI patients with two or more morbidities had a greater risk of death and had an all-cause mortality twice that of non-SMI patients, similar to previous evidence [37–44].

### Strengths and limitations

Our assessment of multimorbidity and mortality was strengthened through access to a large patient population database and a lengthy follow-up of 11 years. Most people with an SMI are likely to have been diagnosed and treated within the hospital system, rather than primary care, and are unlikely to be missed on the administrative records. Given that the inclusion of non-SMI people hospitalised with a physical health condition provides a population of non-healthy controls, mortality rates of people with SMI compared to the general population are likely to be even higher than presented here. Unlike other studies, we examined multiple life-limiting conditions, enabling a more precise examination of the cumulative effect of multimorbidity on mortality in individuals with SMI, all adjusted for multiple confounders. The large population size also allowed us to separately examine both schizophrenia and bipolar disorder as outcomes. However, the use of hospital data means that we were unable to adjust for various key confounders such as diet, smoking and physical activity, or severity of illness. Additionally, we had no data on dates of first onset of physical health conditions, but only dates of hospital contact. Specific cause of death was unavailable at the time of study; we can only assume that cause of death in most cases was related to the life-limiting conditions that we explored.

## Data Availability

The linked administrative data that support the findings are safe-guarded and only available to members of the research team. However, these data can be accessed by other researchers through the Honest Broker Service on submission of the usual approval application forms. Syntax files developed to produce findings reported in this study are available on request from the corresponding author.
